# Matrix Metallopeptidase 9 Promotes Contraction in Human Uterine Myometrium

**DOI:** 10.1007/s43032-024-01778-3

**Published:** 2025-01-08

**Authors:** Craig C. Ulrich, Lauren L. Parker, Janet A. Lambert, Lexa Baldwin, Iain L. O. Buxton, Neda Etezadi-Amoli, Normand Leblanc, Heather R. Burkin

**Affiliations:** 1 Department of Pharmacology, University of Nevada, Reno School of Medicine, 1664 North Virginia St., Reno, NV 89557 USA; 2 Department of Obstetrics and Gynecology, University of Nevada, Reno School of Medicine, 1664 North Virginia St., Reno, NV 89557 USA

**Keywords:** Human reproduction, Labor, Myometrium, Pregnancy, Uterus

## Abstract

Matrix metallopeptidase 9 (MMP9) is a secreted zinc-dependent peptidase known for extracellular remodeling. MMP9 is elevated in tissues from women experiencing preterm labor, and previous research has shown that the addition of combined matrix metallopeptidases 2 and 9 (MMP2/9) enhances uterine contractions. We hypothesized that adding MMP9 alone would enhance myometrial contractions and that specific MMP9 inhibition would suppress uterine contractions. In myometrial tissue from women undergoing term Caesarean sections, we observed an increased contractile response as measured by area under the curve over time in tissues treated with MMP9 compared to vehicle-treated controls (*p* = 0.0003). This effect was primarily due to increased contraction frequency in MMP9-treated tissues compared to controls (*p* < 0.0001). Specific inhibition of MMP9 with the highly selective MMP9 inhibitor 1 (AG-L-66085) reduced contractile responses in myometrial tissues from pregnant women. We observed a reduction in the oxytocin-induced contractile response as measured by area under the curve over time (*p* < 0.0001) and contraction amplitude (*p* < 0.0068) in AG-L-66085-treated tissues compared to vehicle-treated controls. To determine the effects of MMP9 inhibition in the absence of exogenous oxytocin, we tested the effects of AG-L-66085 on spontaneous contractions. The area under the curve (*p* = 0.0415) and amplitude (*p* = 0.0354) of spontaneous contractions were reduced in response to 1 μM AG-L-66085, and the inhibitory effects increased as the AG-L-66085 concentration increased. Together, these data support the hypothesis that elevated MMP9 promotes myometrial contractions and labor, while its inhibition promotes relaxation.

## Introduction

Matrix metallopeptidases (MMPs) are zinc-dependent proteases that are involved in several key processes during pregnancy [1–3]. MMP9 (formerly known as gelatinase B) is produced and secreted as an inactive 92 kDa zymogen that undergoes catalytic cleavage to produce an 83 kDa active enzyme. The MMP9 enzyme contains zinc and calcium binding motifs, fibronectin type II-like repeats, and a hemopexin domain that modulates substrate selectivity and specificity [3].

MMP2 and MMP9 have long been known for their roles in extracellular matrix breakdown and remodeling, as well as in promoting human cervical ripening in preparation for labor [2–4]. MMP9 is highly expressed by neutrophils and inflammatory macrophages and plays a major role in promoting uterine angiogenesis and vascularization to support placental development and function during pregnancy. MMP9 also facilitates angiogenesis in endometrial and other cancers and altered expression is associated with a number of uterine pathologies [5].

Recent evidence suggests a specific role for MMPs in the regulation of uterine contractions during parturition. Addition of MMP2/9 inhibitors at the peak of an oxytocin-induced contraction immediately increased “bursts” of contractile activity in virgin and pregnant rat myometrium, suggesting MMPs might act via novel mechanisms in this tissue [6]. These findings prompted our laboratory to examine the effects of MMP2/9 on contractions in uterine tissues from pregnant women. Surprisingly, inhibition of MMP2/9 reduced the contractile response in human uterine tissue in a sustained, dose-dependent, and reversible manner [7]. In contrast, addition of physiologically relevant concentrations of purified MMP2/9 increased the contractile force over time. Finally, systemic inhibition of MMP2/9 via SB-3CT delayed parturition in mice [7]. The differential effects on rat vs human tissues may be attributed to the experimental time course, species, and/or pregnancy state [7]. Together, these results indicate that, at physiologically relevant concentrations as reported in tissues and fluids from normal and pathological conditions of pregnancy [8], the combination of MMP2/9 enhances the oxytocin-induced contractile response over time in human uterine tissue and supports the hypothesis that levels of these molecules can affect birth timing.

In the mouse uterus, MMP9 transcript decreases as normal pregnancy progresses and rises again near term, while MMP2 expression remains low in late pregnancy [9]. MMP9 activity is elevated in the amniotic fluid of patients undergoing term or preterm spontaneous rupture of membranes and parturition [8, 10, 11]. MMP9 concentrations are also elevated in serum from women experiencing either term or preterm labor compared to gestational age-matched controls [12, 13]. Finally, polymorphisms in the MMP9 gene have been associated with preterm premature rupture of membranes and recurrent spontaneous abortion [14–16]. We have shown that MMP9 is elevated in preterm laboring uterine myometrial tissue and, therefore, is locally available to affect the contractile response [7]. Together, these observations led us to hypothesize that elevation of MMP9 is sufficient to increase contractile responses in the pregnant human uterus and that specific inhibition of this enzyme would promote relaxation. We performed experiments to determine specific effects of MMP9 on uterine contractions.

## Materials and Methods

### Participants

Uterine tissue from the upper edge of the transverse incision in the lower uterine segment was obtained from twenty-seven women with informed consent and with the University of Nevada, Reno’s Institutional Review Board (IRB) approval. Eligible participants were pregnant individuals who were at least 37 weeks gestation undergoing caesarean sections in the absence of HIV or hepatitis infection at Renown Regional Medical Center, Reno, NV from June 2021-June 2024. Medical history data was collected and de-identified. Patient ages ranged from 24–43 years (mean age 32 years). Study participants were 74% Caucasian, 18.5% Hispanic, 3.7% Asian, and 3.7% Native American. All were singleton pregnancies with a mean gestational age of 38 weeks (range 37–40 weeks), parity 1–6, with fetal sex 52% female, 48% male. Please see Table 1 for a description of Caesarean section indications.

**Table 1:** Study participant characteristics

Experiment	GA*	Parity	Age	Ethnicity	Fetal Sex	Indication for C-section
Figure 1	39	2	unknown	Caucasian	female	Previous c-section
	40	1	29	Caucasian	female	Breech
	39	2	37	Caucasian	male	Failure to progress
	39	2	34	Asian	male	Breech; choroid plexus cyst
	39	2	38	Caucasian	female	Previous c-section, fibroids
	39	2	38	Caucasian	female	Infection, pre-eclampsia
Figure 2	39	2	27	Caucasian	female	Previous c-section
	37	unknown	35	Caucasian	female	Previous c-section
	38	3	27	Hispanic	male	Previous c-section
	37	3	29	Caucasian	male	Previous c-section
	39	4	29	Caucasian	female	Breech, macrosomia, low lying placenta
	39	4	37	Hispanic	male	Previous c-section
	39	2	28	Hispanic	male	Previous c-section
	37	1	36	Caucasian	female	IUGR, velamentous cord insertion
	37	3	32	Caucasian	male	Previous c-section, gestational hypertension
Figure 3	37	4	30	Caucasian	male	Gestational hypertension
	39	2	24	Caucasian	male	HELLP, morbid obesity, thrombocytopenia
	39	2	27	Native American	female	Previous c-section
	39	1	34	Caucasian	female	Breech
	37	1	32	Hispanic	male	Gestational hypertension
	38	3	30	Caucasian	female	Previous c-section
	38	1	32	Hispanic/Caucasian	female	Breech, IUGR
	39	1	32	Caucasian	female	Previous c-section, rheumatoid arthritis
Figure 4	39	6	27	Caucasian	female	Previous c-section, failure to progress
	39	1	39	Caucasian	male	Breech, suspected macrosomia
	39	4	43	Caucasian	male	Previous c-section
	37	3	38	Caucasian	male	Previous c-section, gestational hypertension
Mean	38	2	32			
Range	37–40		24–43			

^*^Gestational Age

### Myography

Uterine tissue was immediately transported to the laboratory in buffer (120 mM NaCl, 5 mM KCl, 0.587 mM KH_2_PO_4_, 0.589 mM NaH_2_PO_4_, 2.5 mM MgCl_2_, 20 mM glucose, 0.5 mM CaCl_2_, 25 mM Tris, 5 mM NaHCO_3_, pH 7.4) and myometrial samples dissected into 10 × 1x1 mm strips as described [17]. Myometrial strips were suspended in tissue organ baths (DMT 820MO, Ann Arbor, MI) filled with 8 mL Krebs solution (118 mM NaCl, 4.75 mM KCl, 1.2 mM KH_2_PO_4_, 25 mM NaHCO_3_, 20 mM glucose, 1.2 mM MgCl_2_, 1.8 mM CaCl_2_, pH 7.4) bubbled with 95% O_2_; 5% CO_2_ at 37 °C. Strips were mounted longitudinally, with one end fixed and the other end connected to a force transducer; changes in isometric tension, including baseline contractile responses, were recorded as described [17]. The maximum force of the transducer is 30 g (~ 300 mN) with a resolution of 0.01 mN. Strips were maintained under 2–3 g basal tension for at least 30 min and then primed with 60 mM potassium chloride (KCl), followed by washes in Krebs buffer. After spontaneous contractions developed, uterine strips were stimulated with 10 nM oxytocin for 20 min, followed by the addition of activated MMP9 to 1 μg/ml (~ 10^–8^ M) (Sino Biological, Wayne, PA) or vehicle control (PBS, pH 7.4). Results from triplicate strips were recorded for each patient and the effects on uterine contractions were compared to those of triplicate vehicle controls.

In a second series of experiments, after the initiation of spontaneous contractions as described above, uterine strips were stimulated with 10 nM oxytocin for 30 min followed by the addition of 10^‑6^ M AG-L-66085 (MMP-9 Inhibitor I, Santa Cruz Biotechnology, Santa Cruz, CA) or DMSO as the vehicle control.

Next, spontaneous contractions were established as described above but in the absence of exogenous oxytocin, and the baseline response was recorded for at least 30 min, followed by the addition of 10^‑6^ M AG-L-66085 (Santa Cruz Biotechnology, Santa Cruz, CA) or DMSO as the vehicle control.

Finally, AG-L-66085 was applied to spontaneously contracting tissue strips in increasing concentrations ranging from 3 × 10^–7^ to 3 × 10^–6^ M to establish a dose response.

We assessed peak amplitude, interval, and area under the curve [17]. Amplitude was defined as the difference between the highest and lowest force measurements recorded for a given contraction. Contractions were defined as changes reaching a maximum force of at least 25% of the maximum force within the treatment window for the purpose of analyses. Interval between contractions was determined by the difference in seconds between the peak contraction amplitude and the peak amplitude of the previous contraction. Area under the curve was calculated for the entire treatment window (20 or 30 min) using LabChart 8 software with a rolling baseline to account for baseline drift (DMT, Ann Arbor, MI). Data from each strip were normalized to the mean of its baseline measurements (prior to addition of MMP9 or AG-L-66085) and expressed as a ratio (mean treatment: mean baseline).

### Statistical Analyses

**Fig. 1:**
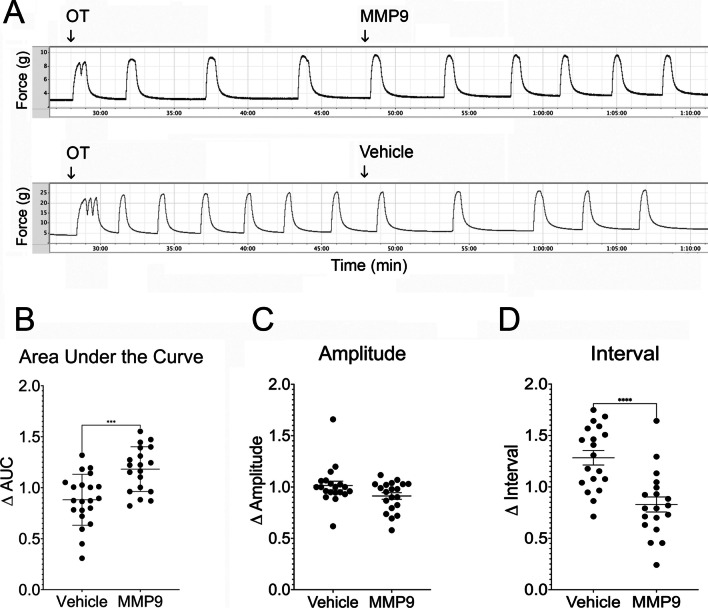
MMP9 enhances oxytocin-induced contractions in ex vivo human uterine smooth muscle. **A** Human term non-laboring myometrial strips were stimulated with oxytocin (OT) and baseline contractions (g force) were recorded. MMP9 was added to 1 µg/mL (~ 10 nM). Control strips were treated with vehicle. **B** Addition of MMP9 increased the area under the curve by 18% compared to an average decrease of 12% in vehicle-treated tissue (***p = 0.0003). **C** Contraction amplitude not significantly different after MMP9 treatment compared to vehicle treatment; however, (**D**) the median interval was shorter after addition of MMP9 (73% of baseline). In comparison, the median interval increased in vehicle-treated tissues (138% of baseline, **** *p* < 0.0001). Data points represent average measurements from tissue samples (*n* = 3 strips per participant × 6 participants = 18 samples per group)

**Fig. 2:**
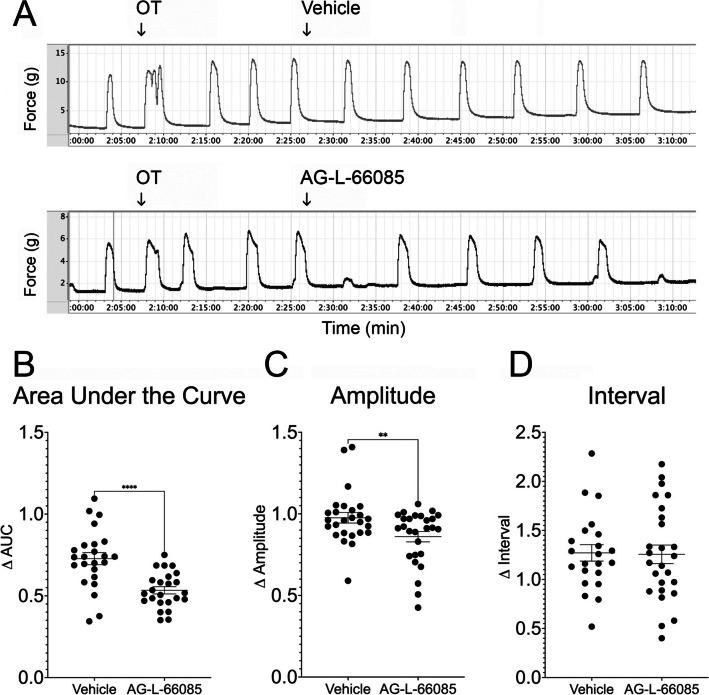
Specific inhibition of MMP9 reduces oxytocin-induced contractions in human uterine smooth muscle. **A** Term non-laboring human myometrial strips were stimulated with oxytocin (OT) and baseline contractions recorded. The MMP9-specific inhibitor AG-L-66085 was added to 1 µM, followed by washout to remove drug effects. Control strips were treated with vehicle only. **B** AG-L-66085 reduced the area under the curve (AUC) by 47% over 15 min compared to 27% in vehicle-treated tissue (****p < 0.0001). **C** Contraction amplitude was reduced by 14% after AG-L-66085 treatment compared to 3.4% after vehicle treatment (**p < 0.0068). **D** MMP9 inhibition did not impact contraction interval. Data points represent average measurements from tissue samples (*n* = 2–4 tissue strips per participant × 9 participants = 23 samples per group)

**Fig. 3:**
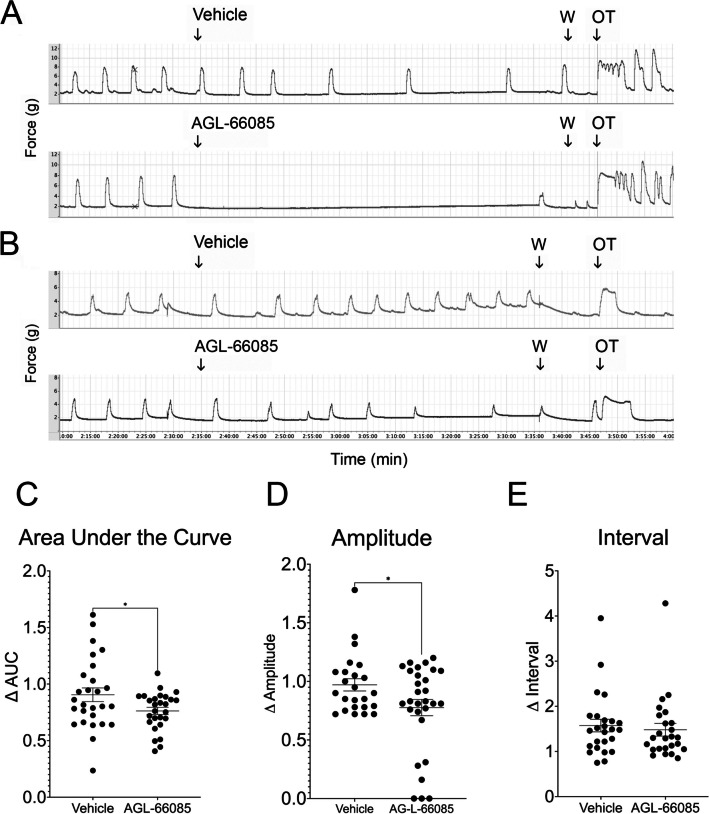
Specific inhibition of MMP9 reduces spontaneous contractions in human uterine smooth muscle. Term non-laboring human myometrial strips were primed with 60 mM KCl for 3 min, followed by washout (W). Baseline spontaneous contractions were recorded for at least 30 min before addition of 1 μM AG-L-66085, control strips were treated with vehicle only. Contractions were recorded for an additional 60 min before washout. **A** Two tissue samples responded to AG-L-66085 with an almost complete loss of contractions. **B** Six tissue samples showed less response to the inhibitor. **C** MMP9 inhibition reduced the area under the curve (AUC) by 24% over 60 min compared to 9% in vehicle-treated tissue (*p = 0.0415). **E** Amplitude was reduced after AG-L-66085 addition (*p = 0.0354), while the interval was unchanged (**D**) in the six tissue samples for which these measurements could be determined. Data points represent average measurements from tissue samples (*n* = 2–4 tissue strips per participant × 8 participants = 24 samples per treatment group)

Statistical analyses were performed using Prism version 10.0.1 (GraphPad, Boston, MA). Groups that met assumptions of normality were compared by Student’s t-test (area under the curve). The Mann–Whitney test was performed to compare datasets with non-normal distributions (amplitude and interval, Figs. 1C, 1D, 2D, 3E). The IC_50_ for AG-L-66085 was determined using the four-parameter variable slope method.

## Results

### Addition of MMP9 Enhances the Oxytocin-Induced Contractile Response in Pregnant Human Myometrial Tissue

To test the hypothesis that specific inhibition of MMP9 reduces uterine contractions, human uterine smooth muscle strips were stimulated with 10 nM oxytocin for 20 min followed by addition of 1 µg/mL (~ 10 nM) purified MMP9 (Fig. 1A). We observed an 18% increase in the contractile response as measured by area under the curve over time in tissue treated with MMP9 compared to a 12% reduction in the vehicle (PBS, pH 7.4) treated tissue (*p* = 0.0003, Fig. 1B). We did not observe a significant change in contraction amplitude in MMP9-treated samples compared to vehicle treated samples (Fig. 1C); however, the contraction frequency nearly doubled in MMP9-treated tissues (i.e., contraction interval decreased, *p* < 0.0001, Fig. 1D). The median interval decreased after MMP9 treatment (from a baseline of 4.1 min between contractions to 2.6 min after MMP9 addition). In comparison, the median interval increased from 3.7 min to 4.6 min after vehicle addition (Fig. 1D).

### Specific Inhibition of MMP9 Reduces the Contractile Response in Pregnant Human Myometrial Tissue Treated with Oxytocin

To test the hypothesis that specific inhibition of MMP9 reduces oxytocin-induced uterine contractions, we tested the ability of the MMP9 blocker AG-L-66085 to reduce myometrial contractions (Fig. 2A). AG-L-66085 was chosen for these experiments because it is a selective, cell permeable, and reversible MMP9 inhibitor with no reported effects on MMP2 [18]. In tissues pre-treated with oxytocin, we observed a 47% reduction in the contractile response as measured by area under the curve over time in tissue treated with MMP9 inhibitor compared to a 27% reduction in the vehicle treated controls (Fig. 2B, *p* < 0.0001). In addition, we observed a 14% reduction in contraction amplitude (g) compared to a 3.4% reduction in vehicle-treated control tissues (Fig. 2C, *p* < 0.0068). Finally, the effect of MMP9 inhibition on contraction interval was not significant. The observed effects were reversible after AG-L-66085 washout as tissues responded to re-stimulation with 10 nM oxytocin.

### Specific Inhibition of MMP9 Attenuates Spontaneous Contractions in Pregnant Human Myometrial Tissue in a Dose-Dependent Manner

**Fig. 4:**
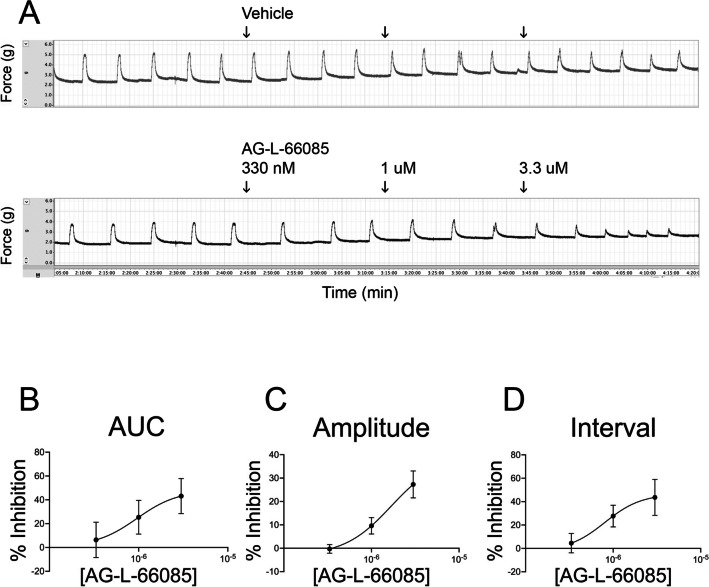
AG-L-66085 reduces spontaneous contractions in a dose-dependent manner. Term non-laboring human myometrial strips were primed with 60 mM potassium chloride (KCl) for 3 min, followed by washout. Baseline spontaneous contractions were recorded for at least 30 min. AG-L-66085 was added to 333 nM, 1 µM, and 3.3 µM for 30 min at each concentration. Control tissue strips were treated with the corresponding vehicle dilution at each dose time point. Contractions were recorded for an additional 30 min before washout. Percent inhibition was calculated by determining the change in response to AG-L-66085 minus any change in response to vehicle. AG-L-66085 inhibited contractions as measured by area under the curve, amplitude, and interval in a dose dependent manner. *n* = 3 tissue strips per participant × 4 participants = 12 samples per treatment group

To determine whether AG-L-66085 would affect spontaneous myometrial contractions, we added the inhibitor to KCl-primed tissues in the absence of oxytocin. The response to AG-L-66085 was highly variable. AG-L-66085 abolished contractions in tissues from two patient samples tested (Fig. 3A), and contraction amplitude, interval, and AUC could not be determined for this subset. The drug had an inhibitory effect in six additional uterine samples (Fig. 3B). Overall, AG-L-66085 reduced the average area under the curve by 24% over 60 min compared to 9% in vehicle-treated tissue (Fig. 3C, *p* = 0.0415). In the subset of six patients for which amplitude and interval could be measured after AG-L-66085 treatment, we observed a reduction in amplitude (Fig. 3D, *p* = 0.0354), but no difference in interval. AG-L-66085-treated tissues remained viable as evidenced by the capacity to respond to stimulation with oxytocin after inhibitor washout (Figs. 3A and B). The effects of AG-L-66085 were dose-dependent with an IC_50_ of approximately 1 μM in our system (Fig. 4). This is comparable to concentrations previously used to observe effects in cells [19].

## Discussion

We have shown that MMP9 is sufficient to promote uterine contractions, while specific MMP9 inhibition attenuates contractions in human uterine tissues in the presence and absence of exogenous oxytocin. In these experiments, addition of purified MMP9 significantly increased contraction frequency, but not amplitude, while inhibition with AG-L-66085 reduced the amplitude of both spontaneous and oxytocin-induced contractions but did not significantly affect the contraction interval. The reasons for this are not clear but might be due to incomplete recovery of contraction-associated machinery between large or frequent contractions. Specific mechanisms may be differentially affected by AG-L-66085 inhibition of endogenous MMP9 activity vs. the addition of exogenous MMP9. Finally, others have reported that MMP9 activity is elevated in women undergoing term or preterm spontaneous rupture of membranes and parturition [7, 8, 10–13]. Together, these data support the hypothesis that elevation of MMP9 contributes to the physiology of labor.

MMP9 is known for its ability to cleave extracellular matrix proteins such as collagen and gelatin to promote tissue remodeling; however, MMP9 produces acute effects on human myometrial contractions, suggesting mechanisms other than extracellular matrix remodeling are involved. Considerable evidence indicates MMPs can act on numerous transmembrane, secreted, and intracellular targets [20, 21]. Increasing evidence indicates MMP9 can interact with cell surface receptors to affect signaling pathways and that secreted MMP9 can be taken up by cells to act on intracellular targets that include cytoskeletal proteins [20, 22].

One possible mechanism by which MMP9 might exert pro-contractile effects is via modulation of intracellular calcium. Oxytocin receptor binding activates inositol triphosphate (IP3) production in myometrium, which triggers calcium release from the sarcoplasmic reticulum (SR) [23, 24]. The resulting plasma membrane depolarization stimulates voltage-dependent calcium channels (VDCCs) to allow further calcium influx [25]. This positive feedback loop results in “waves” of intracellular calcium transients, which are thought to determine contractile force [26]. In response, calmodulin is activated, resulting in phosphorylation and of myosin light chain kinase, which in turn activates the contractile machinery [27]. Interestingly, MMP9 can alter the function of cyclic-nucleotide gated channels and ion channels in other cell types [28–30]. These data suggest a potential role for MMP9 modulation of intracellular calcium or calcium handling within uterine smooth muscle.

Reduction in intracellular calcium is required for subsequent relaxation between contractions, and calcium reuptake via sarcoplasmic/endoplasmic reticulum calcium ATPases (SERCA) contribute to this process [31, 32]. SERCA2 is upregulated in laboring human myometrium and SERCA inhibition reduced the spontaneous contraction interval in laboring but not nonlaboring tissues, suggesting SERCA activity may help regulate contraction frequency during labor [32]. This activity is more pronounced in the absence of functional large-conductance calcium-dependent potassium channels (BK_Ca_), as observed in term myometrium [32, 33]. Loss of MMP9 in cardiomyocytes of diabetic mice reduces contractility by a mechanism that appears to include increased SERCA2 activity and altered calcium transients [34]. Others have confirmed MMP9 downregulation is associated with increased SERCA2a [35]. This observation makes SERCA2 an attractive candidate for MMP9 modulation in myometrium, as the observed heterogeneous effects of AG-L-6608 on spontaneous contractions in term myometrial tissues could be due to differing endogenous levels of SERCA2 and/or BK_Ca_ channel expression or activity [32].

MMP9 may also exert effects on junctional integrity in the uterine myometrium. MMP9 is predicted to cleave the gap junction protein Connexin-43 (Cx43) [36]. Depending on the cell or tissue type, MMP9 may either reduce or enhance Cx43 expression and function [36, 37]. Since Cx43 gap junctions allow ion flow to facilitate excitation coupling, any MMP9-mediated changes that affect gap junction stability or function would be expected to promote coordinated myometrial contractions [36, 38].

MMP9 might also exert pro-contractile effects by affecting inflammation-associated pathways. Both term and preterm labor are characterized by the upregulation of inflammatory signaling in the myometrium [39, 40]. Inflammatory cytokines stimulate upregulation of prostaglandins, gap junction-associated proteins, and oxytocin receptors to transform the myometrium from a quiescent to a contractile state [41, 42]. Many of these cytokines are known MMP9 targets, including IL1β, TNFα, interleukin 8, and transforming growth factor beta 1 (TGFβ1) [43–47]. Production of MMP9 and cytokines can lead to a positive feedback loop to amplify pro-labor signals. In pregnant macaques, IL1β upregulates both TNFα and prostaglandin production, resulting in preterm contractions that are reversible with prostaglandin inhibition [44, 48]. Acute exposure to IL1β and TNFα augments contractile responses in other cell and tissue types [49, 50]. Similarly, IL-8 enhances prostaglandin production and uterine contractile effects of IL1 in nonpregnant rabbits [51]. In murine fibroblasts and airway cells, MMP9 promotes activation of TGFβ1 and contractions while MMP9 inhibition or knockdown reduces these responses [52]. Thus, MMP9 has the potential to mediate several mechanistic pathways that connect inflammation and contractile response.

Our data are consistent with the hypothesis that addition of MMP9 to levels previously reported in human term laboring pregnancy tissues enhances contractile responses and that this effect is not reliant on exogenous oxytocin. We have also shown that specific inhibition of MMP9 attenuates contraction responses in a dose-dependent manner. These data add to our mechanistic understanding of the regulation of labor in women and provide a foundation for future experiments to determine new mechanisms by which this peptidase can modulate uterine contractions.

## Data Availability

Upon publication all data will be available via Dyad at 10.5061/dryad.pzgmsbctx.

## References

[1] Chen J, Khalil RA. Matrix metalloproteinases in normal pregnancy and preeclampsia. Prog Mol Biol Transl Sci. 2017;148:87–165. 10.1016/bs.pmbts.2017.04.001. 28662830 10.1016/bs.pmbts.2017.04.001 PMC5548443

[2] Geng J, Huang C, Jiang S. Roles and regulation of the matrix metalloproteinase system in parturition. Mol Reprod Dev. 2016;83(4):276–86. 10.1002/mrd.22626. 26888468 10.1002/mrd.22626

[3] Nikolov A, Popovski N. Role of gelatinases MMP-2 and MMP-9 in healthy and complicated pregnancy and their future potential as preeclampsia biomarkers. Diagnostics (Basel). 2021;11(3):480. 10.3390/diagnostics11030480. 33803206 10.3390/diagnostics11030480 PMC8001076

[4] Stygar D, et al. Increased level of matrix metalloproteinases 2 and 9 in the ripening process of the human cervix. Biol Reprod. 2002;67(3):889–94. 10.1095/biolreprod.102.005116. 12193399 10.1095/biolreprod.102.005116

[5] Nothnick WB. Regulation of uterine matrix metalloproteinase-9 and the role of microRNAs. Semin Reprod Med. 2008;26(6):494–9. 10.1055/s-0028-1096129. 18951331 10.1055/s-0028-1096129 PMC4082683

[6] Yin Z, et al. Increased MMPs expression and decreased contraction in the rat myometrium during pregnancy and in response to prolonged stretch and sex hormones. Am J Physiol Endocrinol Metab. 2012;303(1):E55-70. 10.1152/ajpendo.00553.2011. 22496348 10.1152/ajpendo.00553.2011 PMC3404560

[7] Ulrich CC, et al. Matrix metalloproteinases 2 and 9 are elevated in human preterm laboring uterine myometrium and exacerbate uterine contractilitydagger. Biol Reprod. 2019;100(6):1597–604. 10.1093/biolre/ioz054. 30951583 10.1093/biolre/ioz054 PMC6561860

[8] Maymon E, et al. Evidence of in vivo differential bioavailability of the active forms of matrix metalloproteinases 9 and 2 in parturition, spontaneous rupture of membranes, and intra-amniotic infection. Am J Obstet Gynecol. 2000;183(4):887–94. 10.1067/mob.2000.108878. 11035332 10.1067/mob.2000.108878

[9] Lombardi A, et al. Expression of matrix metalloproteinases in the mouse uterus and human myometrium during pregnancy, labor, and preterm labor. Reprod Sci. 2018;25(6):938–49. 10.1177/1933719117732158. 28950743 10.1177/1933719117732158

[10] Di Ferdinando A, et al. Expression of matrix metalloproteinase-9 (MMP-9) in human midpregnancy amniotic fluid and risk of preterm labor. Clin Exp Obstet Gynecol. 2010;37(3):193–6. 21077523

[11] Locksmith GJ, et al. Amniotic fluid concentrations of matrix metalloproteinase 9 and tissue inhibitor of metalloproteinase 1 during pregnancy and labor. Am J Obstet Gynecol. 2001;184(2):159–64. 10.1067/mob.2001.108860. 11174496 10.1067/mob.2001.108860

[12] Duran-Chavez J, et al. Relationship between metalloproteinase-2 and -9 levels in plasma and vaginal secretion with preterm birth. Eur J Obstet Gynecol Reprod Biol. 2021;261:217–21. 10.1016/j.ejogrb.2021.03.026. 33879349 10.1016/j.ejogrb.2021.03.026

[13] Tency I, et al. Imbalances between matrix metalloproteinases (MMPs) and tissue inhibitor of metalloproteinases (TIMPs) in maternal serum during preterm labor. PLoS ONE. 2012;7(11): e49042. 10.1371/journal.pone.0049042. 23145060 10.1371/journal.pone.0049042 PMC3493509

[14] Barisic A, et al. Matrix metalloproteinase and tissue inhibitors of metalloproteinases gene polymorphisms in disorders that influence fertility and pregnancy complications: a systematic review and meta-analysis. Gene. 2018;647:48–60. 10.1016/j.gene.2018.01.010. 29309889 10.1016/j.gene.2018.01.010

[15] Ferrand PE, et al. A polymorphism in the matrix metalloproteinase-9 promoter is associated with increased risk of preterm premature rupture of membranes in African Americans. Mol Hum Reprod. 2002;8(5):494–501. 10.1093/molehr/8.5.494. 11994547 10.1093/molehr/8.5.494

[16] Pereza N, et al. Matrix metalloproteinases 1, 2, 3 and 9 functional single-nucleotide polymorphisms in idiopathic recurrent spontaneous abortion. Reprod Biomed Online. 2012;24(5):567–75. 10.1016/j.rbmo.2012.01.008. 22406112 10.1016/j.rbmo.2012.01.008

[17] Arrowsmith S, et al. Contractility measurements of human uterine smooth muscle to aid drug development. J Vis Exp. 2018;(131). 10.3791/56639. 10.3791/56639 PMC5841565 29443077

[18] Vandenbroucke RE, Libert C. Is there new hope for therapeutic matrix metalloproteinase inhibition? Nat Rev Drug Discov. 2014;13(12):904–27. 10.1038/nrd4390. 25376097 10.1038/nrd4390

[19] Webb AH, et al. Inhibition of MMP-2 and MMP-9 decreases cellular migration, and angiogenesis in in vitro models of retinoblastoma. BMC Cancer. 2017;17(1):434. 10.1186/s12885-017-3418-y. 28633655 10.1186/s12885-017-3418-y PMC5477686

[20] Cauwe B, Opdenakker G. Intracellular substrate cleavage: a novel dimension in the biochemistry, biology and pathology of matrix metalloproteinases. Crit Rev Biochem Mol Biol. 2010;45(5):351–423. 10.3109/10409238.2010.501783. 20812779 10.3109/10409238.2010.501783

[21] McCawley LJ, Matrisian LM. Matrix metalloproteinases: they’re not just for matrix anymore! Curr Opin Cell Biol. 2001;13(5):534–40. 10.1016/s0955-0674(00)00248-9. 11544020 10.1016/s0955-0674(00)00248-9

[22] Bassiouni W, Ali MAM, Schulz R. Multifunctional intracellular matrix metalloproteinases: implications in disease. FEBS J. 2021;288(24):7162–82. 10.1111/febs.15701. 33405316 10.1111/febs.15701

[23] Ku CY, et al. Oxytocin stimulates myometrial guanosine triphosphatase and phospholipase-C activities via coupling to G alpha q/11. Endocrinology. 1995;136(4):1509–15. 10.1210/endo.136.4.7895660. 7895660 10.1210/endo.136.4.7895660

[24] Somlyo AP, Somlyo AV. Signal transduction and regulation in smooth muscle. Nature. 1994;372(6503):231–6. 10.1038/372231a0. 7969467 10.1038/372231a0

[25] Monga M, et al. Oxytocin-stimulated responses in a pregnant human immortalized myometrial cell line. Biol Reprod. 1996;55(2):427–32. 10.1095/biolreprod55.2.427. 8828850 10.1095/biolreprod55.2.427

[26] Loftus FC, Richardson MJ, Shmygol A. Single-cell mechanics and calcium signalling in organotypic slices of human myometrium. J Biomech. 2015;48(9):1620–4. 10.1016/j.jbiomech.2015.01.046. 25702249 10.1016/j.jbiomech.2015.01.046 PMC4503816

[27] Word RA, Tang DC, Kamm KE. Activation properties of myosin light chain kinase during contraction/relaxation cycles of tonic and phasic smooth muscles. J Biol Chem. 1994;269(34):21596–602. 8063799

[28] Meighan SE, et al. Cyclic nucleotide-gated channel subunit glycosylation regulates matrix metalloproteinase-dependent changes in channel gating. Biochemistry. 2013;52(46):8352–62. 10.1021/bi400824x. 24164424 10.1021/bi400824x PMC4657727

[29] Remacle AG, et al. Matrix Metalloproteinase (MMP) proteolysis of the extracellular loop of voltage-gated sodium channels and potential alterations in pain signaling. J Biol Chem. 2015;290(38):22939–44. 10.1074/jbc.C115.671107. 26283785 10.1074/jbc.C115.671107 PMC4645627

[30] Wiera G, et al. Mechanisms of NMDA receptor- and voltage-gated L-type calcium channel-dependent hippocampal LTP critically rely on proteolysis that is mediated by distinct metalloproteinases. J Neurosci. 2017;37(5):1240–56. 10.1523/JNEUROSCI.2170-16.2016. 28069922 10.1523/JNEUROSCI.2170-16.2016 PMC6596865

[31] Noble K, et al. A review of recent insights into the role of the sarcoplasmic reticulum and Ca entry in uterine smooth muscle. Eur J Obstet Gynecol Reprod Biol. 2009;144(Suppl 1):S11–9. 10.1016/j.ejogrb.2009.02.010. 19285773 10.1016/j.ejogrb.2009.02.010

[32] Tribe RM, Moriarty P, Poston L. Calcium homeostatic pathways change with gestation in human myometrium. Biol Reprod. 2000;63(3):748–55. 10.1095/biolreprod63.3.748. 10952916 10.1095/biolreprod63.3.748

[33] Khan RN, et al. Ca2+ dependence and pharmacology of large-conductance K+ channels in nonlabor and labor human uterine myocytes. Am J Physiol. 1997;273(5):C1721–31. 10.1152/ajpcell.1997.273.5.C1721. 9374660 10.1152/ajpcell.1997.273.5.C1721

[34] Prathipati P, et al. Ablation of matrix metalloproteinase-9 prevents cardiomyocytes contractile dysfunction in diabetics. Front Physiol. 2016;7:93. 10.3389/fphys.2016.00093. 27014091 10.3389/fphys.2016.00093 PMC4791405

[35] Goerg J, et al. Low-dose empagliflozin improves systolic heart function after myocardial infarction in rats: regulation of MMP9, NHE1, and SERCA2a. Int J Mol Sci. 2021;22(11):5437. 10.3390/ijms22115437. 34063987 10.3390/ijms22115437 PMC8196699

[36] De Bock M, et al. Intracellular cleavage of the Cx43 C-terminal domain by matrix-metalloproteases: a novel contributor to inflammation? Mediators Inflamm. 2015;2015: 257471. 10.1155/2015/257471. 26424967 10.1155/2015/257471 PMC4573893

[37] Vermeer PD, et al. MMP9 modulates tight junction integrity and cell viability in human airway epithelia. Am J Physiol Lung Cell Mol Physiol. 2009;296(5):L751–62. 10.1152/ajplung.90578.2008. 19270179 10.1152/ajplung.90578.2008 PMC2681350

[38] Garfield RE, Sims S, Daniel EE. Gap junctions: their presence and necessity in myometrium during parturition. Science. 1977;198(4320):958–60. 10.1126/science.929182. 929182 10.1126/science.929182

[39] Shynlova O, et al. Integration of endocrine and mechanical signals in the regulation of myometrial functions during pregnancy and labour. Eur J Obstet Gynecol Reprod Biol. 2009;144(Suppl 1):S2-10. 10.1016/j.ejogrb.2009.02.044. 19299064 10.1016/j.ejogrb.2009.02.044

[40] Sivarajasingam SP, Imami N, Johnson MR. Myometrial cytokines and their role in the onset of labour. J Endocrinol. 2016;231(3):R101–19. 10.1530/JOE-16-0157. 27647860 10.1530/JOE-16-0157

[41] Lindstrom TM, Bennett PR. The role of nuclear factor kappa B in human labour. Reproduction. 2005;130(5):569–81. 10.1530/rep.1.00197. 16264088 10.1530/rep.1.00197

[42] Menon R, et al. Novel concepts on pregnancy clocks and alarms: redundancy and synergy in human parturition. Hum Reprod Update. 2016;22(5):535–60. 10.1093/humupd/dmw022. 27363410 10.1093/humupd/dmw022 PMC5001499

[43] Augoff K, et al. MMP9: a tough target for targeted therapy for cancer. Cancers (Basel). 2022;14(7):1847. 10.3390/cancers14071847. 35406619 10.3390/cancers14071847 PMC8998077

[44] Baggia S, et al. Interleukin-1 beta intra-amniotic infusion induces tumor necrosis factor-alpha, prostaglandin production, and preterm contractions in pregnant rhesus monkeys. J Soc Gynecol Investig. 1996;3(3):121–6. 10.1177/107155769600300304. 8796819 10.1177/107155769600300304

[45] Gearing AJ, et al. Processing of tumour necrosis factor-alpha precursor by metalloproteinases. Nature. 1994;370(6490):555–7. 10.1038/370555a0. 8052310 10.1038/370555a0

[46] Schonbeck U, Mach F, Libby P. Generation of biologically active IL-1 beta by matrix metalloproteinases: a novel caspase-1-independent pathway of IL-1 beta processing. J Immunol. 1998;161(7):3340–6. 9759850

[47] Van den Steen PE, et al. Neutrophil gelatinase B potentiates interleukin-8 tenfold by aminoterminal processing, whereas it degrades CTAP-III, PF-4, and GRO-alpha and leaves RANTES and MCP-2 intact. Blood. 2000;96(8):2673–81. 11023497

[48] Sadowsky DW, et al. Indomethacin blocks interleukin 1beta-induced myometrial contractions in pregnant rhesus monkeys. Am J Obstet Gynecol. 2000;183(1):173–80. 10.1067/mob.2000.105968. 10920327 10.1067/mob.2000.105968

[49] Murray DR, Freeman GL. Tumor necrosis factor-alpha induces a biphasic effect on myocardial contractility in conscious dogs. Circ Res. 1996;78(1):154–60. 10.1161/01.res.78.1.154. 8603499 10.1161/01.res.78.1.154

[50] Vicaut E, Rasetti C, Baudry N. Effects of tumor necrosis factor and interleukin-1 on the constriction induced by angiotensin II in rat aorta. J Appl Physiol (1985). 1996;80(6):1891–7. 10.1152/jappl.1996.80.6.1891. 8806891 10.1152/jappl.1996.80.6.1891

[51] Khatun S, et al. Interleukin-8 potentiates the effect of interleukin-1-induced uterine contractions. Hum Reprod. 1999;14(2):560–5. 10.1093/humrep/14.2.560. 10100010 10.1093/humrep/14.2.560

[52] Kobayashi T, et al. Matrix metalloproteinase-9 activates TGF-beta and stimulates fibroblast contraction of collagen gels. Am J Physiol Lung Cell Mol Physiol. 2014;306(11):L1006–15. 10.1152/ajplung.00015.2014. 24705725 10.1152/ajplung.00015.2014 PMC4042193

